# The Biogenesis Process of VDAC – From Early Cytosolic Events to Its Final Membrane Integration

**DOI:** 10.3389/fphys.2021.732742

**Published:** 2021-08-12

**Authors:** Anasuya Moitra, Doron Rapaport

**Affiliations:** Interfaculty Institute of Biochemistry, University of Tübingen, Tübingen, Germany

**Keywords:** beta-barrels, chaperones, mitochondria, outer membrane, TOM complex, VDAC

## Abstract

Voltage dependent anion-selective channel (VDAC) is the most abundant protein in the mitochondrial outer membrane. It is a membrane embedded β-barrel protein composed of 19 mostly anti-parallel β-strands that form a hydrophilic pore. Similar to the vast majority of mitochondrial proteins, VDAC is encoded by nuclear DNA, and synthesized on cytosolic ribosomes. The protein is then targeted to the mitochondria while being maintained in an import competent conformation by specific cytosolic factors. Recent studies, using yeast cells as a model system, have unearthed the long searched for mitochondrial targeting signal for VDAC and the role of cytosolic chaperones and mitochondrial import machineries in its proper biogenesis. In this review, we summarize our current knowledge regarding the early cytosolic stages of the biogenesis of VDAC molecules, the specific targeting of VDAC to the mitochondrial surface, and the subsequent integration of VDAC into the mitochondrial outer membrane by the TOM and TOB/SAM complexes.

## Introduction

Most of the outer membrane (OM) proteins in Gram-negative bacteria are membrane-embedded β-barrel proteins that are composed of anti-parallel β-strands forming a barrel shaped hydrophilic pore in the membrane. In eukaryotes, the presence of β-barrel proteins is restricted to the OM of mitochondria and chloroplasts that were derived from prokaryotic ancestors. The assembly of these proteins into their corresponding OM is in each case facilitated by a dedicated protein complex that contains a highly conserved central β-barrel protein termed BamA/YaeT/Omp85 in Gram-negative bacteria, Tob55/Sam50 in mitochondria, and probably OEP80 in plastids (Ulrich and Rapaport, 2015; Gross et al., 2021). These central components are related to each other and belong to the Omp85 superfamily (Gentle et al., 2005). Voltage Dependent Anion-selective Channels (VDACs) are abundant mitochondrial β-barrel proteins (Schein et al., 1976; Colombini, 1979). Their pore is composed of 19 anti-parallel β-strands whereas strands 1 and 19 are in parallel orientation to each other. VDAC, which was previously known as mitochondrial porin, functions as a channel for transport of metabolites, nucleotides, ions, and even small peptides (Benz, 1989). VDACs are found in mitochondria across the spectrum of life, from unicellular yeasts to plants and all higher eukaryotes. Bakers’ yeast (*Saccharomyces cerevisiae*) has two genes encoding VDACs, *POR1* and *POR2*, while higher eukaryotes like humans have at least three isoforms, *VDAC1*, *VDAC2* and *VDAC3* and plants have up to five such genes (Young et al., 2007; Raghavan et al., 2012).

During the evolution of mitochondria from an ancient endosymbiont, most of the organellar genes, including those encoding predecessors of VDACs, were transferred to the nucleus, with the mitochondrial genome retaining the codes for only few key components of the respiratory chain complexes (Gray et al., 1999). VDACs are thus transcribed in the nucleus and translated on cytosolic ribosomes. Then, they need to be targeted to the correct sub-cellular organelle, namely the mitochondria, and ultimately integrated into the mitochondrial OM (MOM) with the help of dedicated import machineries. In this review, we will highlight recent studies that have discovered cytosolic factors associated with newly synthesized VDAC molecules, the elusive mitochondrial targeting information for VDAC, and finally the mechanisms of insertion and integration of VDAC into MOM.

## Early Cytosolic Events of Newly Synthesized VDAC Molecules

**FIGURE 1:**
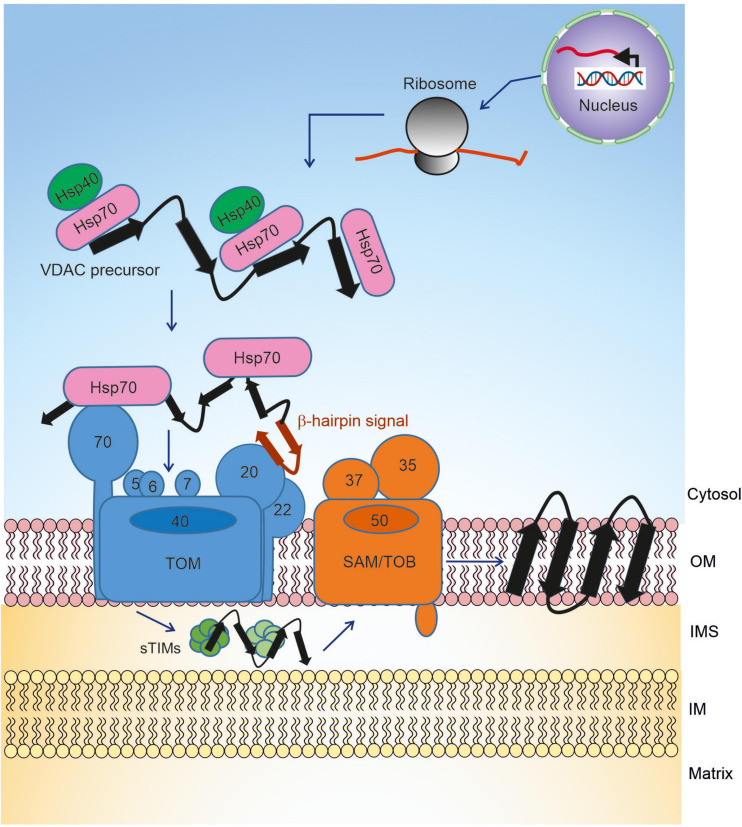
Biogenesis pathway of VDAC. Precursors of VDAC are transcribed in the nucleus, translated on cytosolic ribosomes, and then transported to the mitochondrial surface with the help of chaperones. At the outer membrane, the precursors are initially recognized by receptors of the TOM complex and then translocated across the membrane via the pore formed by Tom40. In the IMS, the small TIM chaperones relay the newly synthesized VDAC molecule to the TOB/SAM complex, which facilitates the final steps of membrane integration.

The first challenge of the biogenesis of VDAC is to keep the newly synthesized molecules in an import competent conformation (Freitag et al., 1982; Rapaport and Neupert, 1999). The rather hydrophobic β-strands that build the transmembrane segments are prone to aggregation in the cytosol. Thus, the newly synthesized VDAC precursors must be bound by cytosolic chaperones to shield these hydrophobic patches, preventing the emerging nascent chain from engaging in unfavorable intra- and inter-molecular interactions (Kim et al., 2013). This association with chaperones maintains them in an import-competent conformation. Recent studies, using yeast as a model system, demonstrate that newly synthesized VDAC molecules dynamically interact with Hsp70 chaperones (Ssa1/2) and their Hsp40 co-chaperones Ydj1 and Sis1 (Figure 1; Jores et al., 2018). Inhibiting the activity of the cytosolic Hsp70 chaperone, preventing its docking to the mitochondrial receptor Tom70, or co-depleting both co-chaperones Ydj1 and Sis1 resulted in a significant reduction in *in vivo* and *in vitro* import of VDAC into yeast mitochondria. Experiments utilizing Hsp70 inhibitors and pull-down assays demonstrated that the interactions between VDAC and Hsp70 chaperones and their physiological role are also conserved in mammalian cells. Moreover, a β-hairpin motif of VDAC, hypothesized to be the mitochondrial targeting signal (see below), was sufficient for the interaction with these (co-) chaperones. It should be emphasized that these (co-) chaperones support the import of not only β-barrel proteins but are also involved in the biogenesis of many additional proteins. Hence, so far, a targeting factor, which is dedicated solely to β-barrel proteins was not identified. The abovementioned chaperones, based on the mitochondrial targeting information, relay the nascent precursors to the receptors of the translocase of the outer membrane (TOM) of mitochondria. Other β-barrel proteins like Tom40 and Tob55/Sam50 appear to follow the same route as VDAC (Jores et al., 2018).

Currently, it is not clear whether the aforementioned cytosolic factors support biogenesis solely by preventing premature unfavorable aggregation or whether they also facilitate specific targeting. The contribution of the chaperone anchor Tom70, located at the mitochondrial surface, to the overall import process suggests that association with chaperones also increases the specificity of organellar targeting.

## Targeting of VDAC to the Mitochondrial Surface

Most mitochondrial precursor proteins contain a cleavable N-terminal presequence that targets them to mitochondria. However, like the other mitochondrial β-barrel proteins, VDAC lacks a cleavable targeting signal. Hence, it remained unclear how the targeting information for VDAC was encoded. Various studies showed that bacterial and chloroplast β-barrel proteins could be targeted and assembled into yeast mitochondria (Walther et al., 2009; Ulrich et al., 2012, 2014). Conversely, VDAC could also be integrated into bacterial outer membranes and form pores there (Walther et al., 2010), suggesting that the targeting information for β-barrel proteins is conserved from bacteria to mitochondria and thus functional in both systems. Since none of the studies could identify a definitive linear amino acid sequence as the targeting signal, it was hypothesized that the targeting signal may be a structural feature of the β-barrel proteins.

Truncation studies showed that the last C-terminal β-strand of mitochondrial β-barrel proteins contains a stretch of amino acids that facilitate their interaction with the TOB/SAM complex. These residues were called the β-signal (Kutik et al., 2008). However, deletion or mutation of the β-signal did not interfere with the initial targeting of newly synthesized β-barrel proteins to mitochondria. Studies involving a bacterial trimeric autotransporter Yersinia adhesion A (YadA), where each subunit contributes four β-strands to a 12-mer β-barrel structure, demonstrated that such proteins can be targeted to mitochondria upon their expression in yeast cells (Müller et al., 2011). This finding implies that even a partial β-barrel structure (like four β-strands) is sufficient for specific mitochondrial targeting. Hence, it was further tested whether a β-hairpin structural motif, which is composed of two β-strands and a loop and represents the most basic repeating structural motif of β-barrel proteins, could be the elusive mitochondrial targeting signal. To support this possibility, it was shown that a peptide corresponding to the last β-hairpin of human VDAC1 could competitively inhibit the *in vitro* import of mitochondrial β-barrels (Jores et al., 2016). Moreover, hybrid proteins of this β-hairpin fused to soluble passenger domains like GFP or DHFR were targeted to mitochondria upon their expression in yeast cells. Such β-hairpin motif has an amphipathic characteristic as eventually, upon its incorporation into a membrane-embedded β-barrel, one phase of the motif will face the lipid core and hence is hydrophobic, whereas the opposite one will be exposed to the pore lumen and thus is rather hydrophilic. Importantly, it was discovered that optimal mitochondrial targeting depends on relative elevated hydrophobicity of those amino acid residues that face the lipid core of the membrane (Jores et al., 2016).

In most eukaryotic cells, mitochondria are the only organelles containing β-barrel proteins. The problem of specific targeting gets an interesting twist in plant cells where plastids can be an alternative destination for such proteins. Klinger et al. (2019) addressed this issue and found that the hydrophobicity is not sufficient for the discrimination of targeting to chloroplasts or mitochondria. By domain swapping between mitochondrial (atVDAC1) and chloroplast (psOEP24) targeted β-barrel proteins, they could demonstrate that the presence of a hydrophilic amino acid at the C-terminus of the penultimate β-strand is also required for mitochondrial targeting. A variant of the chloroplast β-barrel protein psOEP24, which mimics such profile, was efficiently targeted to mitochondria (Klinger et al., 2019).

Collectively, it seems that the combined contribution of several β-hairpin motifs with a highly hydrophobic face assures proper mitochondrial targeting of VDAC.

## Membrane Integration of VDAC by the TOM and TOB/SAM Complexes

Once the chaperone-associated VDAC precursors are targeted to mitochondria via the β-hairpin signal, they interact with the TOM complex at the mitochondrial surface to initiate organellar import (Figure 1). The TOM complex is comprised of the core complex and its peripheral import receptors. The core complex has a central translocon channel, formed by the integral β-barrel protein Tom40, along with several transmembrane accessory proteins namely Tom5, Tom6, Tom7, and Tom22 (Bausewein et al., 2017; Araiso et al., 2019; Tucker and Park, 2019). Tom20 and Tom70 are the receptors involved in the initial recognition of multiple mitochondrial proteins (Neupert and Herrmann, 2007; Figure 1). Several studies hinted at the role of Tom20 in the recognition of β-barrel precursors (Rapaport and Neupert, 1999; Schleiff et al., 1999; Krimmer et al., 2001; Yamano et al., 2008). Using NMR, photo-crosslinking and fluorescence complementation assays, it was recently shown that the β-hairpin element of VDAC interacts with the mitochondrial import receptor Tom20 via the presequence binding region of the latter (Jores et al., 2016). Moreover, direct cross-linking of the β-hairpin motif to Tom70 and the observation that blocking this receptor interferes with the import of VDAC suggested that Tom70 also plays a role in the initial recognition of VDAC (Jores et al., 2016). The involvement of Tom70 can be either via direct recognition of the substrate protein or by serving as a docking site for the chaperone-substrate complex.

Following recognition by the import receptors, the VDAC precursors are translocated across the MOM via the Tom40 channel by interacting with a series of binding sites, probably with increasing affinities (Hill et al., 1998). Upon its emergence at the intermembrane space (IMS), the translocated VDAC molecule interacts with the small chaperones of the translocase of the inner membrane (small TIMs). The IMS chaperone system includes the small Tim proteins, Tim8, Tim9, Tim10, and Tim13 (Koehler et al., 1998). These small chaperones form alternating circular hexamers comprised of three subunits of Tim9 and Tim10, or three subunits of each Tim8 and Tim13 (Webb et al., 2006; Beverly et al., 2008). Site-specific cross-linking indicated that the small TIMs interact with the IMS-exposed part of the N-terminal extension of Tom40 (Shiota et al., 2015).

The small TIMs play an important role in the transfer of the β-barrel precursors of VDAC from the TOM complex to the sorting and assembly machinery (SAM) complex (Hoppins and Nargang, 2004; Wiedemann et al., 2004; Figure 1). The formation of a β-hairpin within the last two C-terminal β-strands of VDAC is crucial for the interaction of the precursors with the TIM chaperones. Structural and mechanistic studies revealed that TIM chaperones hold the VDAC protein precursors in a nascent chain-like extended conformation via multiple clamp-like binding sites (Weinhäupl et al., 2018). Such multiple weak and constantly reshuffling interactions ultimately allow for the efficient release of the precursor to the actual insertase, the SAM complex, which is also known as the topogenesis of outer-membrane β-barrel proteins (TOB) complex (Figure 1; Paschen et al., 2003; Wiedemann et al., 2003; Gentle et al., 2004). The Tim9/10 binding cleft for the β-barrel precursors has conserved hydrophobic residues for these interactions, and mutations in these residues are detrimental to the VDAC biogenesis and overall cell growth.

To facilitate a smooth transfer, the TOM and the TOB/SAM complex can form a super-complex bridged by the cytosolic domain of Tom22 and the peripheral TOB/SAM component, Mas37/Sam37 (Qiu et al., 2013). The core subunit of the TOB/SAM complex is the 16-stranded β-barrel protein Tob55/Sam50, that belongs to the Omp85 superfamily of proteins. Tob55/Sam50 has an N-terminal POTRA domain, which can bind the incoming substrate but is not essential for the β-barrel assembly process. In addition, the TOB/SAM complex harbors two cytosol-exposed peripheral subunits that are involved in formation of a TOM-TOB super-complex (Mas37/Sam37) and stabilization of the TOB/SAM bound form of the precursor (Tob38/Sam35).

**FIGURE 2:**
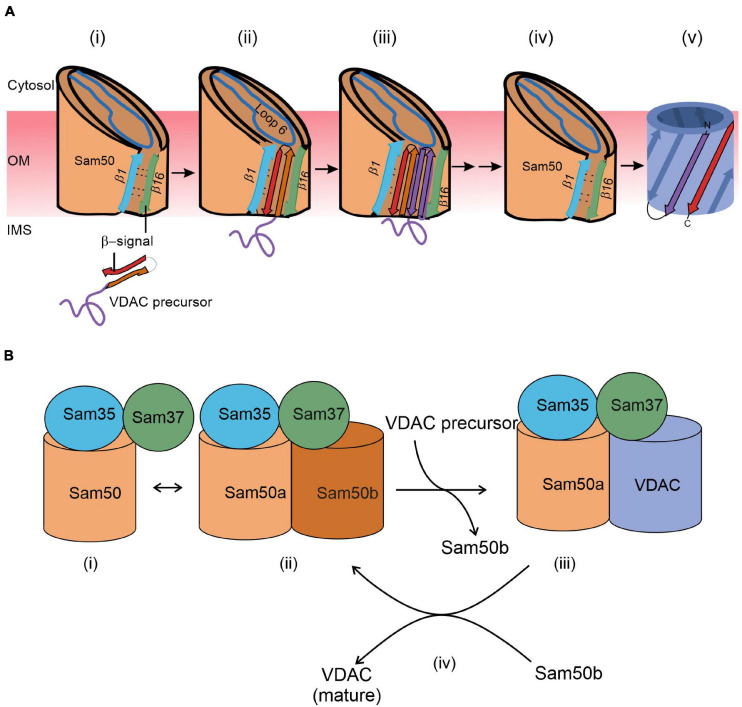
A working model for the final steps of the membrane integration of VDAC. **(A)** Lateral insertion (adapted from Figure 8; Höhr et al., 2018). (i) VDAC precursors approach the outer membrane from the IMS. (ii) The C-terminal β-signal of VDAC precursor interferes with the Sam50 structure by binding to the β1 strand of Sam50 and disrupting the β1–β16 interactions within Sam50. This enforces opening of a lateral gate. (iii) The initial opening is followed by sequential insertion of additional precursor β-hairpins through the lateral gate of Sam50. (iv) The lateral gate of Sam50 re-closes to (v) release the fully formed β-barrel of VDAC into the MOM. **(B)** Barrel switching (based on Takeda et al., 2021). In its substrate-free state, the SAM complex is in equilibrium between the “monomeric” state consisting of Sam50a, Sam35, and Sam37 (i) and a “dimeric” species that contains a second (Sam50b) barrel (ii). The gradually formed VDAC barrel displaces Sam50b (iii). Finally, the fully folded VDAC molecule dissociates from the complex to be replaced by Sam50b (iv).

Our understanding of the final steps in the biogenesis of the VDAC β-barrel precursors evolved dramatically in the last 5 years. Structural studies indicate the formation of a lateral gate between β-strands 1 and 16 of Sam50. Accordingly, and supported by intensive cross-linking assays, the lateral gate insertion model was put forward. This model suggests that the C-terminal β-signal of the precursor initiates opening of the gate by exchange with the endogenous Sam50 β-signal. In addition, loop 6 of Sam50 was found to be crucial for the VDAC precursor transfer to the lateral gate (Höhr et al., 2018). An increasing number of β hairpin–like loops of the precursor insert and fold sequentially and accumulate at the lateral gate (Figure 2A). Finally, hydrogen bonds are formed between the first and last β-strand to close the newly folded VDAC β-barrel. Upon folding at Sam50, the full-length newly formed β-barrel protein is laterally released into the outer membrane and the Sam50 lateral gate closes (Figure 2A). The opening of the putative lateral gate obtained further support from a recent report on the atomic structure of the SAM complex (Diederichs et al., 2020). Membrane thinning in the vicinity of the lateral gate can further facilitate insertion of the β-barrel protein into the lipid bilayer.

The membrane integration model recently obtained a new twist from structural studies. Based on detailed atomic structure of the SAM complex, the barrel swapping model envisions the SAM complex as formed by a SAM monomer (Sam50a along with Sam35 and Sam37) and a Sam50b second barrel (Figure 2B; Takeda et al., 2021). The precursor protein β-signal binds Sam50a as in the lateral gate insertion model. Then, the folded VDAC β-barrel slowly displaces Sam50b and takes its place. Sam37 that originally also interacts with Sam50b, gets gradually involved in interactions with the newly formed VDAC barrel (Figure 2B). Finally, this barrel dissociates from the SAM complex and is integrated into the MOM.

Of note, most of our current knowledge regarding the biogenesis of β-barrel proteins is based on biochemical and structural studies on fungal elements. While the atomic structure of the mammalian TOM complex appears to be rather similar to its fungal counterpart (Wang et al., 2020), not much is known about the SAM complex in higher eukaryotes. It is rather clear that the mammalian Sam50 is the central component of the complex. However, the precise functions of Metaxins1/2/3, which are homologous to yeast Sam35 and Sam37, is not clear yet.

## Perspectives

Our understanding of the factors and machineries involved in the assembly of VDAC proteins into the MOM has made tremendous progress in the last 20 years. We now have detailed atomic structures of the membrane-embedded TOM and SAM complexes, and the hexamer of the small TIM chaperones that transfer the substrate from the former to the latter. Challenges for the future include the characterization of the mammalian SAM complex and to decipher how the various biogenesis steps of VDAC are regulated and adapted to the cellular physiological conditions. Moreover, it will be interesting to determine if after its insertion into the OM, oligomerization, additional folding, or post-translational modifications are necessary for VDAC to become fully functional.

## Author Contributions

AM and DR wrote the manuscript. Both authors contributed to the article and approved the submitted version.

## Conflict of Interest

The authors declare that the research was conducted in the absence of any commercial or financial relationships that could be construed as a potential conflict of interest.

## Publisher’s Note

All claims expressed in this article are solely those of the authors and do not necessarily represent those of their affiliated organizations, or those of the publisher, the editors and the reviewers. Any product that may be evaluated in this article, or claim that may be made by its manufacturer, is not guaranteed or endorsed by the publisher.
